# Dr. B. M. Walker’s Cure of Umbilical Hernia

**Published:** 1878-09-20

**Authors:** 


### DR. B. M. WALKER'S CURE OF UMBILICAL HERNIA.

The ingenuity displayed by Dr. Walker in devising a Truss to meet the requirements of his case of umbilical hernia, as reported in our last, is worthy of special mention. No better evidence of a skillful and intelligent physician or surgeon than the faculty of meeting unlooked for emergencies by a ready and skillful invention of means and appliances adapted to the case.

